# Naringenin Improves Innate Immune Suppression after PRRSV Infection by Reactivating the RIG-I-MAVS Signaling Pathway, Promoting the Production of IFN-I

**DOI:** 10.3390/v15112172

**Published:** 2023-10-29

**Authors:** Jiaying Yu, Haitao Shi, Ke Song, Yuxin Yang, Xinmiao Li, Luyuan Peng, Bendong Fu, Pengfei Yi

**Affiliations:** College of Veterinary Medicine, Jilin University, Changchun 130062, China; yujiaying21@mails.jlu.edu.cn (J.Y.); shiht19@mails.jlu.edu.cn (H.S.); songke20@mails.jlu.edu.cn (K.S.); yangyux22@mails.jlu.edu.cn (Y.Y.); xinmiao22@mails.jlu.edu.cn (X.L.); pengly@jlu.edu.cn (L.P.); fubendong96@163.com (B.F.)

**Keywords:** naringenin, PRRSV, innate immune suppression, RIG-I-MAVS

## Abstract

Porcine reproductive and respiratory syndrome (PRRS) has been prevalent for nearly forty years since it was first reported. It has been one of the major diseases jeopardizing the healthy development of the world swine industry, as well as causing great economic losses to the industry’s economic development. Furthermore, no way has been found to combat the disease due to the immunosuppressive properties of its pathogen porcine reproductive and respiratory syndrome virus (PRRSV) infection. We previously examined the mRNA expression of IFN-I in PRRSV-infected Marc-145 cells at different time periods using qRT-PCR, and found that the mRNA expression of IFN-I in the late stage of PRRSV infection showed suppression. Naringenin is a flavonoid found in citrus fruits and has a very wide range of pharmacological activities. Therefore, the aim of the present study was to investigate the modulatory effect of naringenin on the suppressed innate immune response after PRRSV infection. The expression of IFN-I, IL-10, and ISGs in the late stage of PRRSV infection was examined using qRT-PCR, and the results showed that naringenin improved the expression of antiviral cytokines suppressed by PRRSV infection. Further results showed that naringenin treatment significantly up-regulated the expression of proteins related to the RIG-I-MAV immune signaling pathway, and that naringenin could not significantly activate the RIG-I-MAVS signaling pathway after the addition of the RIG-I inhibitor Cyclo. Overall, these data demonstrated that naringenin could improve the innate immune response suppressed by PRRSV infection by modulating the RIG-I-MAVS signaling pathway. Therefore, our study will provide a theoretical basis for the development of naringenin as a drug against immunosuppressive viral infectious disease infections.

## 1. Introduction

Porcine reproductive and respiratory syndrome (PRRS) is a highly contactable and virulent infectious disease caused by a porcine reproductive and respiratory syndrome virus (PRRSV) infecting swine herds, and is typically characterized by respiratory disease in piglets and reproductive disorders in sows [1]. After the first report in 1987 in the United States, outbreaks of unknown high fever and abortion storms have occurred in Europe and the United States. In 1995, PRRSV was introduced into China, leading to epidemics and spreading to date [2].

In the face of domestic environmental pressure, as well as continuous immunization pressure, the genetic sequence of PRRSV has been mutating continuously, and, at present, the vast majority of PRRSV-2 strains in China can be categorized into spectrum 1 (NADC30-like), spectrum 3 (QYYZ-like), spectrum 5 (VR2332-like), and spectrum 8 (JXAl-like) [3]. NADC30-like strains are mainly characterized by a high mutation rate and potential risk of carrying the virus, and had been found in Beijing, Tianjin, Hebei, Henan, Shandong, Shanxi, Fujian, Zhejiang, Jiangsu, Sichuan, Hubei, and Guangdong by 2015 [4]. The highly pathogenic strain of spectrum 8 is also widely prevalent in various regions of China, and is mainly characterized by high fever, high morbidity, and high mortality in piglets. The morbidity rate of piglets infected with PRRSV is as high as 100% and the mortality rate can be more than 50%, while the abortion rate of sows can be more than 30% [5,6]. The virus has become endemic in most pig breeding areas in the world, causing huge economic losses to the world pig breeding industry and seriously hindering the industry’s sustainable and healthy development [7,8].

PRRSV is a small, capsular single-stranded positive-stranded unsegmented RNA virus with a genome size of approximately 15.4 Kb [9,10,11]. The PRRSV genome structure consists of approximately 15,000 nucleotides encoding at least 10 Open Reading Frames (ORFs), with ORF1α and ORF1b accounting for approximately four-fifths of the full length of the entire genome and being responsible for encoding two large nonstructural polyproteins, which are subsequently hydrolyzed by proteolytic enzymes into approximately 16 nonstructural proteins. Eight ORFs (2α, 2b, 3–7, and 5α) are responsible for encoding the structural GP2α, E, GP3, GP4, GP5α, GP5, M and nucleocapsid (N) proteins [12].

The pathogen has evolved a variety of immune escape mechanisms in response to significant environmental and immune stress. PRRSV infection is strictly cytophilic and predominantly infects monocyte-macrophages (porcine alveolar macrophages) [13]. First, it enters porcine alveolar macrophages through its infection of the essential cell receptor CD163 for self-replication [14,15] and uses a variety of mechanisms to inhibit or evade the innate immunity of the host [16]. Through various non-structural proteins (NSPs) produced in the process of self-replication, the host innate immune system is damaged via degrading, interfering with, or blocking protein–protein signaling in the signaling pathways of the host innate immune system and post-translational modification of some signaling proteins. This inhibits the production of interferon and interferon-stimulated genes [17,18] and delays the host adaptive immune response, resulting in the emergence of neutralizing antibodies at a late stage, which are not capable of neutralizing the virus in a timely manner. Viral particles released from macrophages reach all parts of the body with the bloodstream and colonize immune organs for replication, especially in the thymus and lymph nodes [10,19], where PRRSV infection induces apoptosis in a large number of thymocytes, destroying the immune function of thymocytes and further inducing systemic infections. More fatally, the N protein of PRRSV first induces the emergence of non-neutralizing antibodies, which not only fails to neutralize the virus, but instead promotes further infection of the monocyte-macrophage cell line by the virus and exacerbates the viral infection [20]. This has also been a problem in clinics that are scrupulous about PRRSV vaccine protection, creating a risk of virulence returning to live weakly attenuated vaccines in production practice and inactivated vaccines failing to provide a good level of protection [21,22]. Thus, the immunosuppressive nature of PRRSV infection is a key factor for its successful survival in the host, and the innate immune system is the primary defense against invasion by pathogenic microorganisms. Therefore, we believe that the control of PRRS should focus more on the innate immunosuppressive properties of this viral infection and re-establish the defense barrier of the infected animal body.

The innate immune response is critical for defense against viral infections. Pattern recognition receptors (PRRs) are a class of biomolecules localized in the cell membrane and cytoplasm that are responsible for recognizing viral nucleic acids or viral replication intermediates [23], and upon detection of a viral infection, they activate a natural and undifferentiated immune response mechanism to exert an anti-infective effect. One of the RNA recognition receptors, RIG-I, localized in the cytoplasm, is expressed in essentially all nucleated cells and shows no tissue specificity, suggesting a general role for RIG-I in monitoring viral infections [24]. RIG-I is mainly responsible for recognizing the RNA structure of 5’-PP or 5’-PPP [25]. Normally, in the absence of any pathogenic microbial infection, the RIG-I structure is in a closed-loop-like state, and the activation mode is initiated when the RNA structure of the virus is promptly monitored. With the help of the CTD region of its structure, it recognizes the RNA structure of the virus, followed by the recognition and binding of the RIG-I-CARD structural domain to its junction protein, the mitochondrial antiviral protein MAVS, which activates the downstream effector molecule, TBK1. In turn, this recruits and activates IRF3, which is further phosphorylated into the nucleus, inducing the production of large quantities of IFN-I and pro-inflammatory cytokines [26]. IFN-I establishes the antiviral status of uninfected cells in advance through cellular autocrine or paracrine secretion in order to achieve antiviral infection. However, it has been reported that Nsp1α and Nsp1β can directly inhibit the synthesis of type I interferons or interfere with their signaling pathway, thereby enhancing viral pathogenicity. PRRSV nsp2 antagonizes IFN-β production by inhibiting IRF3 phosphorylation and nucleation. Nsp4 impairs antiviral effects by site-cleaving RIG-I-MAVS, and overexpression of nsp11 down-regulates intracellular MAVS and RIG-I mRNA and protein expression [27]. It is evident that PRRSV has evolved multiple strategies to interfere with host innate immune signaling pathways and achieve immune escape. Therefore, there is an urgent need for a drug that can modulate innate immunity at the early stage of PRRSV infection to break the innate immunosuppressive property of its infection, so that adaptive immunity can also perform its immune function normally.

Naringenin is a flavonoid that is most abundant in citrus fruits [28]. In recent years, there has been increasing interest in the biological functions and pharmacological effects of naringenin, which has been found to have a wide range of biological activities. These include not only promoting carbohydrate metabolism, and increasing antioxidant defense and scavenging of oxygen free radicals [29], but also having a very good effect in antiviral infections. One study found that naringenin inhibited ZIKV infection of human A549 cells in a concentration-dependent manner, acting at the stage of viral replication or assembly of viral particles [30]. In addition, naringenin inhibits HCV secretion and prevents the assembly of infectious virus particles [31]. Studies on hepatitis C virus, influenza A virus, dengue virus, and Zika virus have further demonstrated that Nar treatment may be a promising strategy for suppressing viral replication and infection [32].

More notably, naringenin has also been included in therapeutic studies of effective drugs for COVID-19. Naringenin not only exerts a therapeutic effect on COVID-19 by inhibiting its major protease, 3 chymotrypsin-like protease (3CLpro) [33], and by decreasing the activity of angiotensin-converting enzyme receptors [34,35,36], but it may also modulate the effects of human coronavirus infections by targeting the molecular target, TPC2 [37,38,39]. Naringenin also activates interferon-stimulated response elements and enhances IFN-I production by increasing IRF7 expression [40] and NK cell activity by enhancing NKG2D ligand expression [41].

Therefore, this study investigated whether naringenin can improve this immunosuppression by modulating the RIG-I signaling pathway from the perspective of the innate immune response suppressed by PRRSV infection. The aim was to achieve the purpose of anti-infection, to provide a theoretical basis for the prevention and treatment of immunosuppressed viral infections, and, at the same time, to lay a solid foundation for the development of drugs based on naringenin.

## 2. Materials and Methods

### 2.1. Reagents, Cells, and Virus

Naringenin (purity > 99%) was purchased from Chengdu Must Bio-Technology Co.,Ltd., Chengdu, China. Cyclo (Phe-Pro) was purchased from MCE, Shanghai, China. RIG-I/DDX58 Polyclonal antibody, MAVS;VISA Polyclonal antibody, TBK1 Polyclonal antibody, IRF3 Polyclonal antibody (ProteinTech Group, Wuhan, China), P-IRF3 Polyclonal antibody (ProteinTech Group, Wuhan, China), and anti-GAPDH antibody (ImmunoWay Biotechnology Co., Beijing, China) were used in the study.

Marc-145 cells (African green embryonic kidney epithelial cell line, ATCC) were cultured in Dulbecco’s modified Eagle’s medium (DMEM; Sigma-aldrich, St. Louis, MO, USA) containing 10% fetal bovine serum (FBS; Gibco, Thermo Fisher Scientific, Waltham, MA, USA) at 37 °C in a humidified atmosphere containing 5% CO_2_. This approach supports PRRSV replication in vitro and is commonly used in laboratories. 

The PRRSV-JL/07/SW strain was gifted by the Laboratory of Animal Infectious Diseases, College of Animal Medicine, Jilin University. The viral titer was 10^6.25^ TCID_50_/0.1 mL via the endpoint dilution method. The PRRSV-JL/07/SW strain was utilized for all experiments and is represented by “Virus” in this article.

### 2.2. Cell Viability Analysis

Cell viability was determined by the CCK8 (Sunbao Biotech, Shanghai, China) method. Marc-145 cells were inoculated in 96-well plates at a cell density of 8 × 10^3^ per well and incubated at 37 °C, 5% CO_2_ for 12 h. After the medium was replaced with DMEM containing 2% FBS, different concentrations of naringenin were prepared (0–50 μg/mL) and continued to incubate in the incubator until 48 h. A quantity of 10 μL of CCK8 reagent was added to each well and incubated at 37 °C with 5% CO_2_ for 45 min. Absorbance was measured at 450 nm using an enzyme marker.

### 2.3. In Vitro Infection with PRRSV

The cultured Marc-145 cells were inoculated into 6-well plates. When the cell density reached about 60%, the cell culture medium was discarded, 1 mL/well of sterile PBS was used to wash the cells 2–3 times, 500 μL of 100TCID50/well of viral volume was used to inoculate the cells, the cells were adsorbed at 4 °C for 2 h, and the viral solution was discarded. To the virus infection group, 2 mL 2% maintenance medium was added to each well, and continued to incubate for 0 h, 6 h, 12 h, 24 h, 36 h, 48 h, 60 h, 72 h, 84 h, and 96 h. Finally, the RNA of the cells at different times of viral infection was extracted, and the mRNA changes of IFN-I at different times of infection were detected using the method of RT-qPCR.

### 2.4. Quantitative Reverse Transcription Polymerase Chain Reaction (RT-qPCR)

According to the manufacturer’s instructions, mRNA was reverse transcribed into cDNA using the reverse transcription kits (TRAN, Beijing, China). SYBR Green Master (ROX) (Roche, Shanghai, China) was used to measure the expression of related genes. The relative expression levels were determined using the 2^−∆∆Ct^ method, with GAPDH mRNA as a reference. See Table 1 for primer details. 

### 2.5. Western Blot

Equal amounts of proteins were separated by SDS polyacrylamide gel electrophoresis (SDS-PAGE), then transferred to PVDF and blocked with 5% skimmed milk powder for 4 h at room temperature and incubated with specific primary antibodies against RIG-I, MAVS, TBK1, P-IRF3, IRF3, and GAPDH overnight at 4 °C. The membranes were then washed three times with TBST, and then with horseradish peroxidase. The membrane was then washed three times with TBST, and then incubated with horseradish peroxidase-conjugated goat anti-mouse/rabbit IgG (H + L) antibody at room temperature for 2 h. Excess antibody was washed with TBST and developed, and the amount of expression of each target protein was analyzed in grayscale using Image J (Fiji v2.15.0) with GADPH as an internal reference.

### 2.6. Statistical Analysis

Data are expressed as mean + standard deviation (SD) (*n* = 3). Analysis of variance (ANOVA) was performed using GraphPad Prism 6.0 software to determine statistical significance between multiple groups. A *p*-value of <0.05 was considered significant.

## 3. Results

### 3.1. The Maximum Safe Concentration Range of Naringenin

**Figure 1:**
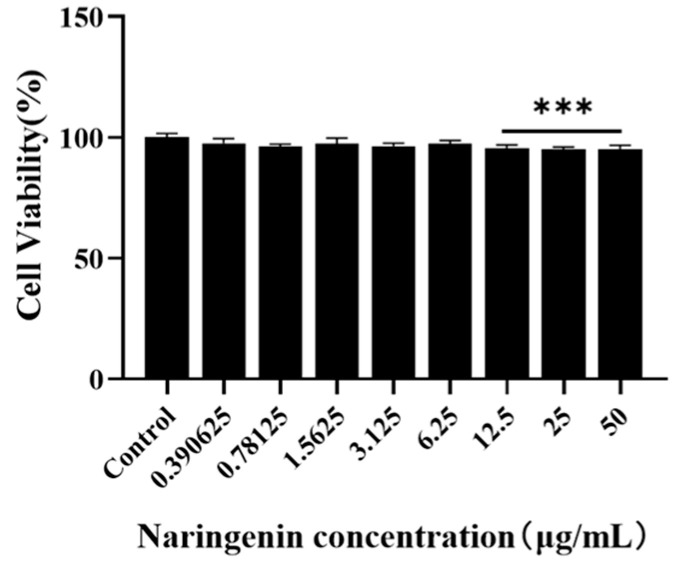
The maximum safe concentration range of naringenin on Marc-145 was detected using the CCK8 method. *** *p* < 0.001, vs. the control group.

Regarding the maximum safe concentration range of naringenin on Marc-145 cells, according to the results of the CCK8 method (Figure 1, naringenin had no obvious toxic effect on Marc-145 cells within 6.25 μg/mL. Thus, the maximum safe concentration of naringenin of 6.25 μg/mL was preliminarily selected as the test concentration for subsequent inhibition of PRRSV infection.

### 3.2. IFN-I mRNA Expression Decreased in the Later Stages of PRRSV Infection

**Figure 2:**
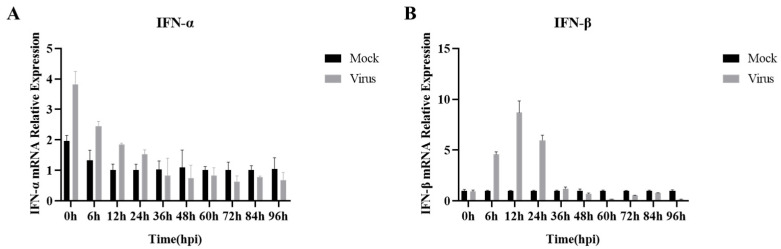
PRRSV infection with mRNA expression of IFN-I at different time periods. (**A**) IFN-α mRNA expression; (**B**) IFN-β mRNA expression. Data are expressed as the mean and SD of three independent experiments.

Previously, in order to investigate the changes in IFN-I mRNA at different times of PRRSV infection, we detected the expression of IFN-I mRNA at 10 time points: 0 h (inoculation with PRRSV at 4 °C, adsorption was completed in 2 h), and 6–96 h of PRRSV infection, via RT-qPCR. The results showed that IFN-α expression (Figure 2A) decreased sequentially with the prolongation of PRRSV infection in a time-dependent manner. From 36 h of PRRSV infection, IFN-α mRNA expression showed inhibition. In contrast, the mRNA expression of IFN-β (Figure 2B) in the pre-infection period of PRRSV (0–12 h) increased sequentially with the prolongation of PRRSV infection until it peaked at 12 h of infection. Subsequently, as the infection continued, PRRSV kept replicating, the mRNA expression of IFN-β decreased sequentially, and the PRRSV infection at 48 h began to show inhibition. Overall, IFN-I mRNA expression was elevated during the pre-infection period of PRRSV and decreased during the post-infection period, with the strongest inhibition of IFN-I mRNA expression at 96 h of viral infection.

### 3.3. Naringenin Up-Regulates the Expression of Relevant Immune Cytokines Suppressed after PRRSV Infection

**Figure 3:**
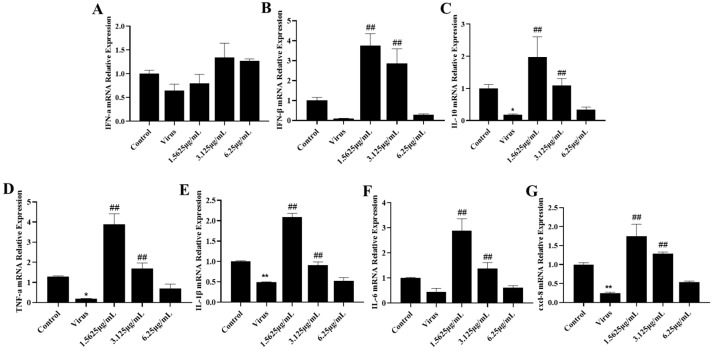
Naringenin up-regulates the expression of immune cytokines. (**A**) IFN-α mRNA expression; (**B**) IFN-β mRNA expression; (**C**) IL-10 mRNA expression; (**D**) TNF-α mRNA expression; (**E**) IL-1β mRNA expression; (**F**) IL-6 mRNA expression; (**G**) cxcl-8 mRNA expression. Data are expressed as the mean and SD of three independent experiments. ** *p* < 0.01, * *p* < 0.05, vs. the virus group. ## *p* < 0.01, vs. the control group.

We detected the expression levels of IFN-a, IFN-β, and IL-10 mRNA in cells using qRT-PCR. According to the results of IFN-I mRNA expression levels (Figure 3A,B), the PRRSV-infected group down-regulated the expression levels of IFN-a and IFN-β genes, and significantly suppressed the expression of IL-10 mRNA (Figure 3C), compared with the cells in the uninfected blank group. While different concentrations of naringenin increased the expression levels of IFN-I and IL-10 mRNA to different degrees, the drug concentration of 3.125 μg/mL had the best effect. Further, in order to investigate the changes in relevant inflammatory and immune cytokines after PRRSV infection, the expression levels of TNF-α, IL-1β, IL-6, and cxcl-8 mRNA in cells were detected by qRT-PCR (Figure 3D–G), and the results showed that compared with the cells in the uninfected blank group, all of the PRRSV-infected groups down-regulated the expression levels of TNF-α, IL-1β, IL-6, and cxcl-8 mRNA, while both medium and low concentrations of naringenin significantly increased the expression of TNF-α, IL-1β, IL-6, and cxcl-8 mRNA.

### 3.4. Naringenin Up-Regulates the Expression of Interferon-Stimulated Genes Inhibited by PRRSV Infection

**Figure 4:**
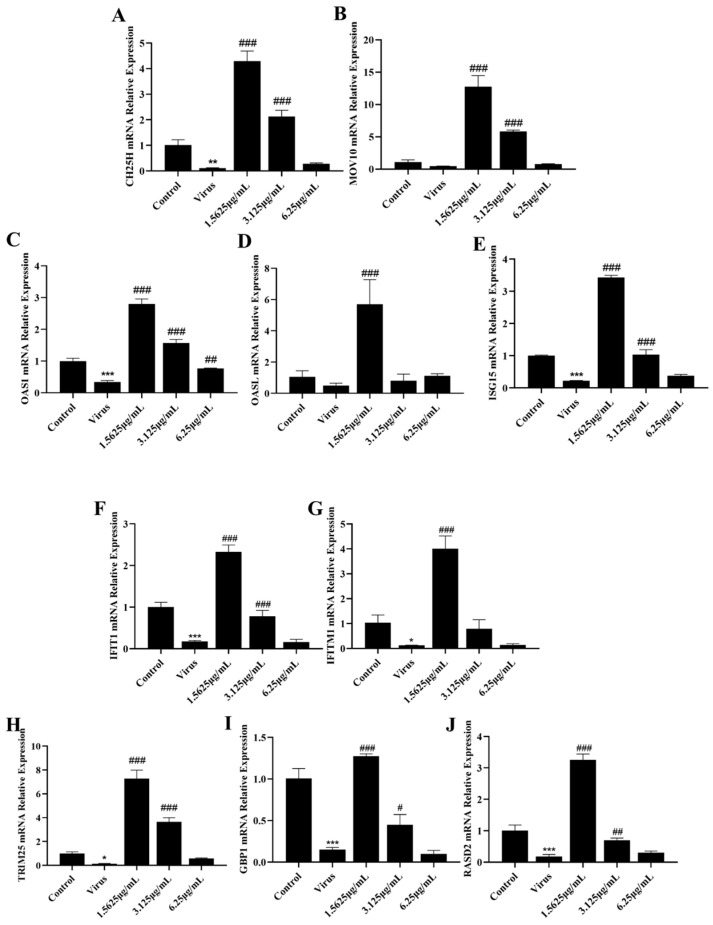
Naringenin up-regulates the expression of ISGs. (**A**) CH25H mRNA expression; (**B**) MOV10 mRNA expression; (**C**) OAS1 mRNA expression; (**D**) OASL mRNA expression; (**E**) ISG15 mRNA expression; (**F**) IFIT1 mRNA expression; (**G**) IFITM1 mRNA expression; (**H**) TRIM25 mRNA expression; (**I**) GBP1 mRNA expression; (**J**) RASD2 mRNA expression. Data are expressed as the mean and SD of three independent experiments. *** *p* < 0.001, ** *p* < 0.01, * *p* < 0.05, vs. the control group. ### *p* < 0.001, ## *p* < 0.01, # *p* < 0.05, vs. the virus group.

However, the antiviral action of interferons also involves a very complex signaling process, and the interferon itself induces the production of a large number of interferon-stimulated genes to exert its antiviral action as an important link in antiviral signaling. Therefore, to investigate whether naringenin further induces the production of relevant interferon-stimulated genes and thus improves the immunosuppressive properties of PRRSV infection, we examined the mRNA expression of relevant interferon-stimulated genes using qRT-PCR. The results showed that the mRNA expression of CH25H, MOV10, TRIM25, ISG15, OAS1, OASL, IFIT1, IFITM1, RASD2, and GBP1 mRNAs in the PRRSV-infected group was significantly lower than that of the mRNAs in the cells of the uninfected group. In addition, different concentrations of naringenin treatment groups all up-regulated the mRNA expression of these interferon-stimulated genes to a certain extent (Figure 4), among which the low concentration of 1.5625 μg/mL of naringenin had the best inducing effect. The effect of 3.125 μg/mL of naringenin was the second most effective, but also had a certain inducing generative effect. Therefore, the above results suggest that at the level of cellular infection, naringenin can improve the inhibitory properties of PRRSV on the interferon system and has some immunomodulatory effects.

### 3.5. Naringenin Reactivates RIG-I-MAVS Innate Immunity Suppressed after PRRSV Infection

**Figure 5:**
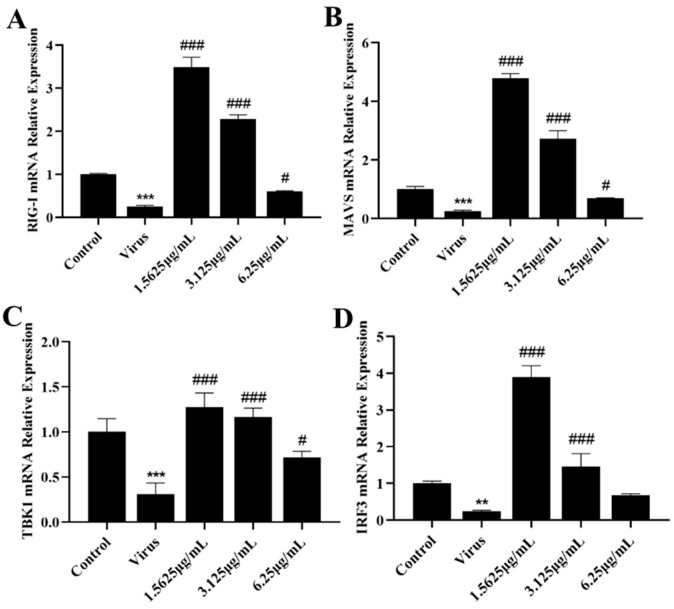
Naringenin up-regulates the expression of genes and proteins related to the RIG-I-MAVS signaling pathway. (**A**) RIG-I mRNA expression; (**B**) MAVS mRNA expression; (**C**) TBK1 mRNA expression; (**D**) IRF3 mRNA expression. Data are expressed as the mean and SD of three independent experiments. *** *p* < 0.001, ** *p* < 0.01, vs. the control group. ### *p* < 0.001, # *p* < 0.05, vs. the virus group.

**Figure 6:**
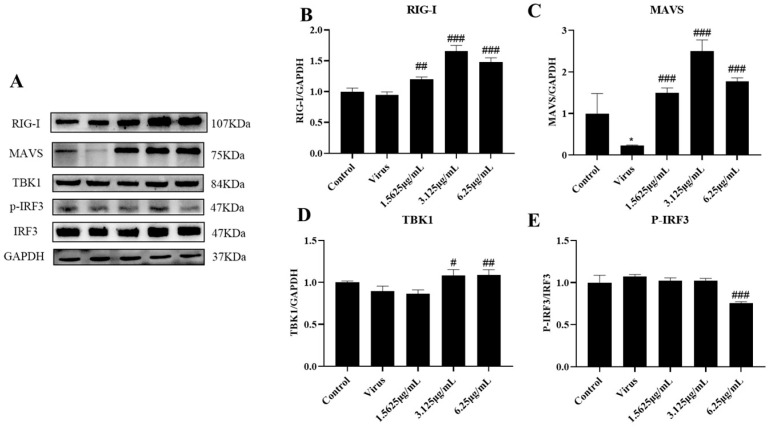
Naringenin up-regulates the expression of genes and proteins related to the RIG-I-MAVS signaling pathway. (**A**) Western blotting analysis of protein expression; (**B**) RIG-I protein expression; (**C**) MAVS protein expression; (**D**) TBK1 protein expression; (**E**) p-IRF3 protein expression. Data are expressed as the mean and SD of three independent experiments. * *p* < 0.05, vs. the control group. ### *p* < 0.001, ## *p* < 0.01, # *p* < 0.05, vs. the virus group.

The innate immune system is the first line of defense against invasion by pathogenic microorganisms, in which RIG-I, a pattern recognition receptor localized in the cytoplasm, recognizes the invasion of PRRSV, which in turn activates downstream effector molecules and induces the expression of IFN-I. However, PRRSV infection has severe immunosuppressive properties. In order to further investigate the specific mechanism by which naringenin ameliorates the immunosuppressive properties of PRRSV infection, we explored the effects of naringenin on the expression levels of factors related to the RIG-I-MAVS signaling pathway of the innate immune RNA recognition receptor at the mRNA level and the protein level using qRT-PCR and WB, respectively. The results showed (Figure 5A–D) that at the late stage of Marc-145 infection with PRRSV, the PRRSV-infected group down-regulated the expression of RIG-I, MAVS, TBK1, and IRF3 mRNA levels compared with the cells of the uninfected group, and the different concentrations of naringenin-treated groups all up-regulated the expression of their mRNAs to a certain extent. In addition, the protein level results showed (Figure 6A–E) that compared with the cells in the uninfected group, the PRRSV-infected group down-regulated the protein expression of RIG-I, MAVS, and TBK1, which significantly down-regulated the protein expression of MAVS. Furthermore, unlike the mRNA results, the PRRSV infection increased the level of phosphorylation of IRF3, which may be related to the cell’s own immunity against infection. Compared with the PRRSV-infected group, different concentrations of naringenin-treated groups all up-regulated the protein expression of RIG-I, MAVS, and TBK1 to some extent.

**Figure 7:**
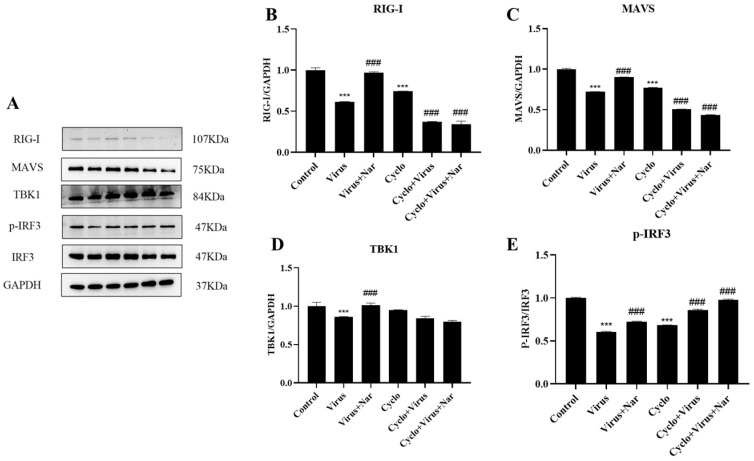
Naringenin exerts innate immunomodulatory effects by modulating the RIG-I-MAVS signaling pathway. (**A**) Western blotting analysis of protein expression; (**B**) RIG-I protein expression; (**C**) MAVS protein expression; (**D**) TBK1 protein expression; (**E**) p-IRF3 protein expression. Data are expressed as the mean SD of three independent experiments. *** *p* < 0.001, vs. the control group. ### *p* < 0.001, vs. the virus group.

To better demonstrate that naringenin could reactivate the RIG-I-MAVS signaling pathway inhibited by PRRSV infection, we performed further validation. The RIG-I immunomodulatory effects of naringenin were further explored at the protein level by the addition of the RIG-I inhibitor Cyclo (Phe-Pro), which specifically interacts with RIG-I and inhibits IRF3 activation. The results showed that the Cyclo (Phe-Pro) group significantly suppressed the expression of the RIG-I protein compared with the uninfected blank group, while the virus-infected group with the addition of Cyclo (Phe-Pro) further down-regulated the protein expression of RIG-I compared with the virus-infected group. However, compared with the virus-infected group, the 6.25 μg/mL naringenin group, to which Cyclo (Phe-Pro) was added along with the virus infection, did not up-regulate the protein expression of RIG-I and MAVS, but instead significantly inhibited the protein expression of RIG-I and MAVS (Figure 7B,C).

For TBK1 protein expression (Figure 7D), there was no significant difference in the Cyclo (Phe-Pro) group compared with the uninfected blank group, and there was no significant difference in the 6.25 μg/mL naringenin group in which the virus was infected with the simultaneous addition of Cyclo (Phe-Pro); however, there was a different degree of reduction in TBK1 protein expression in all of them. Similarly, for IRF3 phosphorylation (Figure 7E), both the viral infection with Cyclo (Phe-Pro) and the 6.25 μg/mL naringenin group with Cyclo (Phe-Pro) added to the viral infection significantly elevated the phosphorylation level of IRF3 compared with the viral infection group. We hypothesize that this is due to the complex network-regulatory nature of the innate immune system, as TBK1 and IRF3 themselves are not only regulated by the RIG-I-MAVS signaling pathway alone, but also receive excitation signals from a wide range of pattern-recognition receptors, such as RLR and cGAS, in order to promote a range of antiviral responses, such as interferon synthesis. Therefore, the above results suggest that naringenin has a good immunomodulatory effect by reactivating the RIG-I-MAVS signaling pathway and ameliorating the innate immunosuppression caused by PRRSV infection.

## 4. Discussion

PRRS, a highly contactable viral infection [42], has been one of the major diseases jeopardizing the health of the global swine industry since it was reported, and has caused serious losses to the global livestock economy in the last four decades. As the genome of this pathogen has a high mutation frequency [43], it further increases its genomic instability in the face of environmental and immunological pressures. In China, it is clear that the vaccination strategy is not effective in controlling PRRSV infection and may even further exacerbate the infection. PRRSV infection exhibits a widespread and strong immunosuppression, and our traditional Chinese herbs have a unique advantage in immunomodulation. The therapeutic concept of Chinese herbs from ancient times to the present is to regulate the balance of yin and yang, to enhance one’s own positive qi, and to repel external evils; as the saying goes, when positive qi exists inside, evils cannot be dried up. Zhengqi in Chinese medicine is similar to immunity in modern medicine. In recent years, research into the immunomodulation of traditional Chinese herbal medicines in China has gradually received attention.

Therefore, in this study, the active compound naringenin was screened from the classical compound formula of Ma Xing Shi Gan Tang with the help of network pharmacology, and was found to be able to inhibit PRRSV infection through a preliminary study. It was further found that naringenin had a wide range of biological effects through a review of the literature. Most studies on naringenin have focused on inflammatory responses, oxidative defenses [44], and the effects of naringenin on viral infectious receptors or the replication phase of viruses from the perspective of this viral infection. There are few studies on naringenin’s biological (antiviral) activity from the perspective of the cellular or innate immunity of the organism itself. Because PRRSV is a single-stranded positive-stranded RNA virus, infection has immunosuppressive properties. Infection with PRRSV in the clinic often exhibits immunosuppressive features such as first-produced non-neutralizing antibodies that promote PRRSV infection, delayed production of neutralizing antibodies, and persistent infection, whereas the immunosuppression of PRRSV infection at the cellular level is mostly reflected in the inhibition of IFN-I expression. Therefore, the present study demonstrated that naringenin, from the perspective of the cellular level, and the innate immunity to RNA recognition receptor RIG-I, ameliorates the immunosuppressive properties of PRRSV infection and restores the expression of IFN-I by reactivating the RNA recognition receptor RIG-I and further promotes the conduction of the RIG-I-MAVS signaling pathway.

The innate immune response is the first line of defense against invasion by pathogenic microorganisms [45], and in order to establish a more stable and persistent infection, PRRSV must first shield the defense mechanism of the innate immune response. PRRSV is a single-stranded positive-stranded RNA virus. During the process of infection of invading cells, the innate immune pattern recognition receptor RIG-I can recognize the viral RNA in the invading cells and connect with the antiviral junction protein MAVS, which is localized in the mitochondria. MAVS is massively aggregated in the mitochondria, and it begins to recruit downstream junction proteins, such as TRADD, TRAF3, and RIP1. It eventually activates the IKK complex, which further activates IRF3 and NF-κB-mediated signaling pathways and induces the expression of type I interferons and pro-inflammatory factors [24,46], which have a very important innate immune effect during viral infection.

However, with the prolongation of PRRSV infection, PRRSV continuously replicates, assembles, and produces a large number of non-structural proteins (NSPs) in the cell. A large number of studies have shown that these nonstructural proteins act in multiple ways and at multiple stages of the innate immune response. In the present study, we found that IFN-I mRNA expression was suppressed from 36–48 h of PRRSV infection to the late stage. Further exploration revealed that PRRSV suppressed not only the mRNA expression of IFN-I, but also the expression of ISGs in the late stage of the infection, and directly inhibited the activation of the RIG-I-MAVS innate immune signaling pathway. All of the different concentrations of naringenin treatment ameliorated this suppression of the innate immune response to some extent, reactivating the RIG-I-MAVS innate immune signaling pathway that had been suppressed by PRRSV infection. Further, by adding the RIG-I inhibitor Cyclo, it was found that after inhibiting the expression of RIG-I, the treatment of naringenin did not significantly elevate the protein expression of RIG-I, MAVS, and TBK1 compared to the virus-infected group. This could indicate that the effect of naringenin in ameliorating the innate immunosuppression of PRRSV infection was mainly achieved by activating the RIG-I-MAVS signaling pathway. However, pattern recognition receptors for innate immunity generally include four classes: toll-like receptors (TLRs), retinoic acid-inducible gene-I (RIG-I)-like receptors (RLRs), nucleotide oligomerization domain (NOD)-like receptors (NLRs), and C-type lectin receptors (CLRs), and a series of intracellular nucleic acid receptors. We have only conducted studies of naringenin on the intracellular RNA recognition receptor RIG-I, and are not certain whether naringenin similarly affects the expression of the other innate immune pattern recognition receptors; additional experiments are needed to verify this. Meanwhile, due to the frequent mutation of the PRRSV genome, a variety of lineage strains have evolved in clinical production. In this paper we chose to explore the fifth lineage (VR2332-like) strain, which does not comprehensively represent all PRRSV infectious strains in clinical production, but the immunosuppressive properties of PRRSV infection are common to all PRRSV strains. However, more spectral strains are still needed to validate the broad spectrum of naringenin with respect to the innate immunosuppressive properties of PRRSV infection. Due to the strict cytophilic nature of PRRSV infection, further validation on primary porcine alveolar macrophages (PAMs) is needed at a later stage.

In summary, naringenin was able to improve the suppressed IFN-I expression after PRRSV infection and reactivate the innate immune RIG-I-MAVS signaling pathway. This finding demonstrates the potential of naringenin to be an effective antiviral drug.

## 5. Conclusions

Our study showed that naringenin reactivated the RIG-I-MAVS signaling pathway, which was suppressed during the late stage of PRRSV infection, and up-regulated the expression of IFN-I and ISGs by targeting RIG-I, an innate immune RNA recognition receptor. These results suggest that naringenin has the potential to be developed as a novel drug to improve immunosuppression of PRRSV infection in the future.

## Figures and Tables

**Table 1:** Sequences of primers.

Primer Names	Primer Sequences (5′-3′)
IL-10-F	GGGTTGCCAAGCCTTGTCTGAG
IL-10-R	CTTGATGTCTGGGTCGTGGTTCTC
IFN-α-F	ACCTTTGCTTTACTGGTGGCC
IFN-α-R	ATCTGTGCCAGGAGCATCAAG
IFN-β-F	TGCTCTCCTGTTGTGCTTCTCC
IFN-β-R	CATCTCATAGATGGTCAATGCGG
CH25H-F	CATCGTGTTCTGCCTGCTACTCTTC
CH25H-R	CGAGGACGGGTTCTGGTGGTG
MOV10-F	GCTCCGCAACTACAGGTCTCATC
MOV10-R	CGATCCACGACATCAGCACAGG
TRIM25-F	CCTGGTGTGTGGAGTGGTTCAAC
TRIM25-R	GCAACAGCGAAGAAGATGACAAAGC
ISG15-F	GGTGGTGGACAGATGCGATGAAC
ISG15-R	CGAAGGTCAGCCAGAACAGGTC
OAS1-F	GCGAGTTCTCCACCTGCTTCAC
OAS1-R	ACTAGGCGGATGAGGCTCTTGAG
OASL-F	CTGTGCGGACCGTGGAGGAG
OASL-R	TGGCTGGGAGTTGGGAAGAGAAG
IFIT1-F	TGAGCCTCCTTGGGTTCGTCTAC
IFIT1-R	GTTCTCAAAGTCAGCAGCCAGTCTC
IFITM1-F	CTCTTCTTGAACTGGTGCTGTCTGG
IFITM1-R	ACTTGGCGGTGGAGGCATAGG
RASD2-F	ACCTTGTCCTGCTGTTCTGCTG
RASD2-R	CCTCTTTGGTCTCATCTGGCTCTC
GBP1-F	CGAGGGTCTGGGAGATGTAGAGAAG
GBP1-R	CCTGCTGGTTGATGGTTCCTATGC
RIG-I-F	CTGACTGCCTCGGTTGGTGTTG
RIG-I-R	CTCCAGTTCCTCCAGGTTGTCTTTG
MAVS-F	AGAGACCAGGTGAGCAAGGGAAG
MAVS-R	GACACAGCAAGAGGCAGAAGGAAG
TBK1-F	AAGCCTTCTGGTGCAATATCTGGAG
TBK1-R	ACCTGAAGACCCCGAGAAAGACTG
IRF3-F	GAGGCTCGTGATGGTCAAGGTTG
IRF3-R	AGTGGGTGGCTGTTGGAAATGTG
GAPDH-F	TGACATCAAGAAGGTGGTGAAGCAG
GAPDH-R	GTGTCGCTGTTGAAGTCAGAGGAG

## Data Availability

The datasets generated for this study are available from the corresponding author upon request.
